# Impact of COVID-19 on Outpatient Care for Urological Conditions at a University Hospital

**DOI:** 10.7759/cureus.29460

**Published:** 2022-09-22

**Authors:** Ana Clara N Spessoto, Fernando Nestor Facio, Luís Cesar F Spessoto

**Affiliations:** 1 Medicine, Medical School of Catanduva, Catanduva, BRA; 2 Surgical Specialties, Medical School of São José do Rio Preto (FAMERP), São José do Rio Preto, BRA

**Keywords:** urology, treatment, diagnosis, clinic, coronavirus

## Abstract

Introduction: The 2019 coronavirus (COVID-19) rapidly spread throughout the world, with diverse negative consequences in all fields, including the continuity of treatment for patients with other diseases. The public hospital for high-complexity procedures affiliated with the São José do Rio Preto School of Medicine, São Paulo, Brazil, normally has a high annual quantity of outpatient appointments. However, the consequences of COVID-19 led to a reduction in the number of appointments, exams, and outpatient procedures. The aim of the present study was to evaluate the impact of the pandemic on outpatient care for individuals with urological conditions at a university hospital.

Patients and Methods: A retrospective, descriptive, cross-sectional study was conducted involving the analysis of all outpatient appointments for individuals with urological conditions at the Urology Clinic of the São José do Rio Preto Hospital, São Paulo, Brazil, between January 2019 (pre-pandemic period) and December 2020 (after the first year of COVID-19). The variables of interest were sex, age, and affected organs of the genitourinary system.

Results: Among the 4,972 outpatient appointments involving patients with urological conditions in 2019, 70.7% involved males and the largest portion of patients were in the seventh decade of life (40.02%). Among the 4,584 outpatient appointments in 2020, 69.9% involved males and the largest portion were in the seventh decade of life (47.07%). Significant differences were found in the number of outpatient appointments between 2019 and 2020 in all age groups (p < 0.0001). The most affected organs of the genitourinary system in both 2019 and 2020 were the prostate (46.07% and 56.31%, respectively), bladder (30.56% and 22.48%, respectively), and kidney/ureter (22.85% and 19.68%, respectively), with no significant differences between the two years.

Conclusion: The COVID-19 pandemic exerted an impact on outpatient care for individuals with urological conditions at a university hospital, leading to a reduction in the number of appointments. No change was found with regard to the sex of the patient. In contrast, an increase was found in the number of patients 60 years of age or older during the year of the pandemic. The most affected organs of the genitourinary system were the prostate, bladder, and kidney/ureter in both years analyzed.

## Introduction

The 2019 coronavirus (COVID-19) that emerged in Wuhan, China [1] spread rapidly throughout the world [2,3]. Diverse negative consequences were felt in all fields, including the continuity of treatment for patients with other diseases. Changes occurred in the understanding of priorities in the health field, as human resources (medical and paramedical staff), materials, drugs and hospital beds in wards and intensive care units were prioritized to provide care for patients infected with COVID-19. Social distancing also contributed to the reduction in appointments, exams, and treatment for patients with other conditions.

Routine urological care was impacted and discussions were necessary on how to provide care for these patients [4]. One initiative in Brazil was Law nº 13.989/2020, which addressed the use of telemedicine during the crisis caused by SARS-CoV-2 [5]. Throughout the world, guidelines were formulated to assist in the determination of which outpatient procedures should be performed and which should be suspended [6-8].

Moreover, residents in urology may have had their education compromised due to the reduction in the number of outpatient appointments, despite the use of telemedicine. Indeed, the changes imposed by the COVID-19 pandemic led to a reduction in the participation of residents in urological activities [9,10].

The university hospital affiliated with the São José do Rio Preto School of Medicine, São Paulo, Brazil, is a high-complexity institution with a large number of annual outpatient appointments. The consequence of COVID-19 led to a reduction in the number of appointments, exams, and outpatient procedures. In this context, investigating the impact of the pandemic on urological outpatient care is justified. Despite the risk of contamination, patients with urological conditions should be seen, provided that safety guidelines and protocols are respected since such patients run the risk of the aggravation of certain clinical conditions.

The aim of the present study was to evaluate the impact of the pandemic on outpatient care for individuals with urological conditions at a university hospital considering possible associations with sex, age, and affected organs of the genitourinary system.

## Materials and methods

A retrospective, descriptive, cross-sectional study was conducted involving the analysis of all outpatient appointments for individuals (irrespective of ethnicity) with urological conditions at the Urology Clinic of the São José do Rio Preto Hospital, São Paulo, Brazil, between January 2019 (pre-pandemic period) and December 2020 (after first year of COVID-19). This study received approval from the Human Research Ethics Committee of the São José do Rio Preto School of Medicine (certificate number: 47083921.0.0000.5415).

Data were collected from the computational system of the hospital. The variables of interest were sex, age, and affected organs of the genitourinary system in patients with urological conditions in 2019 and 2020 (first year of the pandemic).

The data were entered into spreadsheets in the Excel program. Descriptive analysis involved the calculation of frequencies as well as central tendency and dispersion measures. Pearson’s chi-squared test was used for the comparison of frequencies. The IBM SPSS Statistics for Windows, Version 23.0 (Released 2014; IBM Corp., Armonk, New York, United States) and GraphPad InStat 3.10 (Dotmatics, Boston, United States) programs were used for all analyses, with a p-value ≤ 0.05 considered indicative of statistical significance.

## Results

Among the 4,972 outpatient appointments involving patients with urological conditions in 2019, 70.7% involved males and the largest portion of patients were in the seventh decade of life (40.02%). Among the 4,584 outpatient appointments in 2020, 69.9% involved males and the largest portion were in the seventh decade of life (47.07%).

No significant difference was found in the comparison of the sex of the patients with urological conditions in outpatient care between 2019 and 2020 (p = 0.7727, chi-squared test). In contrast, significant differences were found in the number of outpatient appointments between 2019 and 2020 in all age groups (p < 0.0001) (Table 1).

**Table 1:** Distribution of variables according to the quantity of outpatient appointments for individuals with urological conditions in the two years analyzed. * significant difference, chi-squared test

Variables	2019		2020		p
	N	%	N	%	
Sex					
Male	3515	70.7	3203	69.9	0.7727
Female	1457	29.3	1381	30.1	
Age group					
< 20	244	4.91	149	3.25	
> 20<40	9571	9.25	642	14.01	< 0.0001*
> 40<60	1781	35.82	1635	35.67	
> 60	1990	40.02	2158	47.07	

The most affected organs of the genitourinary system in 2019 were the prostate (46.07%), bladder (30.56%) and kidney/ureter (22.85%). No significant reduction or increase occurred in 2020; the most affected organs were also the prostate (56.31%), bladder (22.48%), and kidney/ureter (19.68%) (Table 2).

**Table 2:** Distribution of organs affected according to the quantity of outpatient appointments for individuals with urological conditions in the two years analyzed. Letters and numbers in parenthesis correspond to International Classification of Diseases (ICD)

Variables	2019		2020	
	N	%	N	%
Kidney and/or ureter (N20)	305	22.85	309	19.68
Bladder (C67)	408	30.56	353	22.48
Prostate (N40+C61)	615	46.07	884	56.31
Testicle (N45)	5	0.37	6	0.38
Penis and/or urethra (C60)	2	0.15	18	1.15
Total	3556	100	3093	100

## Discussion

The results of the present study show a reduction in the quantity of outpatient appointments of individuals with urological conditions at a university hospital during the pandemic. The male sex predominated in both the year before and the first year of the pandemic. A reduction occurred in the number of appointments for all age groups, except individuals 60 years of age or older, for whom an increase was found in 2020. Individuals less than 20 years of age accounted for the least number of appointments in the period studied.

In the analysis of the main organs of the genitourinary system, the prostate, bladder and kidney/ureter were the most affected in both years. Patients with prostate conditions accounted for the most appointments, irrespective of the pandemic, possibly due to the fact that such conditions are more prevalent among patients 60 years of age or older [11]. Moreover, the guidelines recommend not interrupting therapy in cases of prostate diseases without medical approval, which justifies appointments of patients in this age group even during the pandemic.

Conditions that affect the bladder require medical care, as symptoms such as hematuria, micturition pain, and urinary retention should be prioritized [12]. In the present study, the quantity of outpatient appointments diminished in the first year of the pandemic, which may be related to patients visiting emergency rooms for care.

Analyzing the quantity of outpatient appointments in patients with conditions of the kidneys and/or ureter, a slight increase was found in 2020, suggesting that these patients maintained clinical follow-up irrespective of the pandemic. Situations such as compromised renal function, fear of invasive procedures or aggravated pain due to a lack of medical follow-up may explain this finding [13,14].

A reduction of approximately 10% was found in the quantity of outpatient appointments for individuals with urological conditions in the first year of the pandemic compared to the previous year. However, some patients may also have been seen at the emergency service of this tertiary university hospital, which is a regional reference center for a population of approximately two million residents (Instituto Brasileiro de Geografia e Estatística, Conjuntura Econômica, 2021).

In the scenario of the pandemic, outpatient appointments for patients with urological conditions should be maintained at tertiary centers that have the necessary infrastructure to deal with complications related to COVID-19 and provide safe specialized care for patients [15]. Moreover, outpatient follow-up of these patients is necessary, since some conditions, such as prostate cancer, may progress due to the situation of the pandemic [15].

## Conclusions

The COVID-19 pandemic exerted an impact on outpatient care for individuals with urological conditions at a university hospital, leading to a reduction in the number of appointments. No change was found with regard to the sex of the patients. In contrast, an increase was found in the number of patients 60 years of age or older during the year of the pandemic. The most affected organs of the genitourinary system were the prostate, bladder, and kidney/ureter in both years analyzed.
